# Altered conformational structures of nervous necrosis virus surface protrusions and free coat proteins after incubation at moderate-low temperatures

**DOI:** 10.1038/s41598-019-45094-2

**Published:** 2019-06-14

**Authors:** Hyun Jung Gye, Toyohiko Nishizawa

**Affiliations:** Department of Aqualife Medicine, Chonnam National University, Yeosu, 59626 Republic of Korea

**Keywords:** Virus structures, Viral pathogenesis

## Abstract

Nervous necrosis virus (NNV) is a pathogenic fish virus belonging to family *Nodaviridae*. The objective of this study was to analyze stabilities of NNV surface protrusion and free coat protein (CP) conformational structures by analyzing changes of NNV infectivity and antigenicity after incubation at moderate-low temperatures. When cultured NNV suspension was incubated at 45 °C, its infectivity declined gradually but its antigenicity maintained. In contrast, both infectivity and antigenicity of purified NNV declined after incubation at 45 °C. After heat-treatment, surface protrusions of NNV particles disappeared completely, although viral particle structures maintained. Therefore, the reduction in NNV infectivity appeared to specifically occur as a result of heat-denaturation of virus surface protrusions. The loss of NNV infectivity in the presence of fetal bovine serum (FBS) was delayed compared to virus heated in the absence of FBS, demonstrating that FBS could function as a stabilizer for conformational structures of NNV surface protrusions. Moreover, the stabilizing function of FBS changed depending on salt concentration. Continued maintenance of antigenicity for heated cultured NNV suspension containing free-CPs may suggest that conformational structures corresponding to protrusion-domain of free-CP are more heat-stable than those of surface protrusions on NNV particles.

## Introduction

Nervous necrosis virus (NNV), a member of the genus *Betanodavirus* in the *Nodaviridae* family, can infect more than 120 fish species and cause high mortality in aquaculture facilities worldwide^1–3^. Several kinds of NNV vaccines have been developed^2,3^ and many can generate NNV-neutralizing antibodies^4–13^. Recently, efficient methods have been developed to induce convalescence in fish after NNV infection^14–19^. Interestingly, NNV-neutralizing antibodies could not be detected in these convalescent fish despite the fact that these fish are strongly protected against re-infection by NNV^20^. This suggests that there might be some slight differences in antigenicity between inactivated and naïve NNV particles.

The crystal structure of NNV particle has been well established using virus-like particles (VLPs) with reverse genetic technology^21–23^. NNV has a non-enveloped spherical shape with a diameter of 25–30 nm. It consists of a single coat protein (CP) with a relative molecular mass (*M*_r_) of 42,000 and two molecules of positive sense single-stranded RNA^24^. NNV CP has three major domains: an N-terminal arm, a shell domain (S-domain), and a protrusion domain (P-domain). Trimeric P-domains can form 60 protrusions on the NNV particle surface^21,22^. These surface protrusions play a crucial role in the antigenicity and receptor interactions during virus infection^22^. Therefore, epitopes for generating NNV-neutralizing antibodies can be located on these protrusions. At least three different serotypes of NNVs have been reported based on neutralization testing using anti-NNV rabbit sera^25–27^, although NNVs are classified into four genotypes based on nucleotide sequences of variable regions of RNA2^28,29^.

The optimum temperature for virus multiplication ranges from 15 °C to 30 °C depending on NNV genotypes and upper limit temperatures range from 32 °C to 35 °C. It has been reported that these temperatures mainly depend on genomic RNA1 which encodes RNA polymerase, although genomic RNA2 coding for CP also plays a role^30–34^. Infectivity of NNV gradually declines at 37 °C. It is drastically curtailed at ≥60 °C^35,36^. This could be due to denaturation of NNV surface protrusions because these protrusions have heat-sensitive conformational structures^37^. They can also be easily denatured by treatment with carbonate/bicarbonate buffer (pH 9.6)^38^, suggesting that they could be particularly unstable under certain conditions. In our preliminary experiments, we observed that infectivity of cultured NNV suspension gradually declined following incubation at a moderate-low temperature, although its antigenicity was maintained. Currently, details of how NNV surface protrusions, CPs, and particle structures are denatured by heat-treatment remain unknown.

Analyzing changes in protein structure and function by treatment at different temperatures is a basic biochemical approach. Thus, the objective of this study was to determine stabilities of NNV surface protrusions and free-CPs by analyzing both infectivity and antigenicity after incubation at moderate-low temperatures. Conformational structures were visualized by scanning electron microscopy. Influence of fetal bovine serum (FBS) on stability of NNV surface protrusions was also investigated.

## Results

### Infectivity and antigenicity of cultured NNV suspensions after incubation at different temperatures

**Figure 1:**
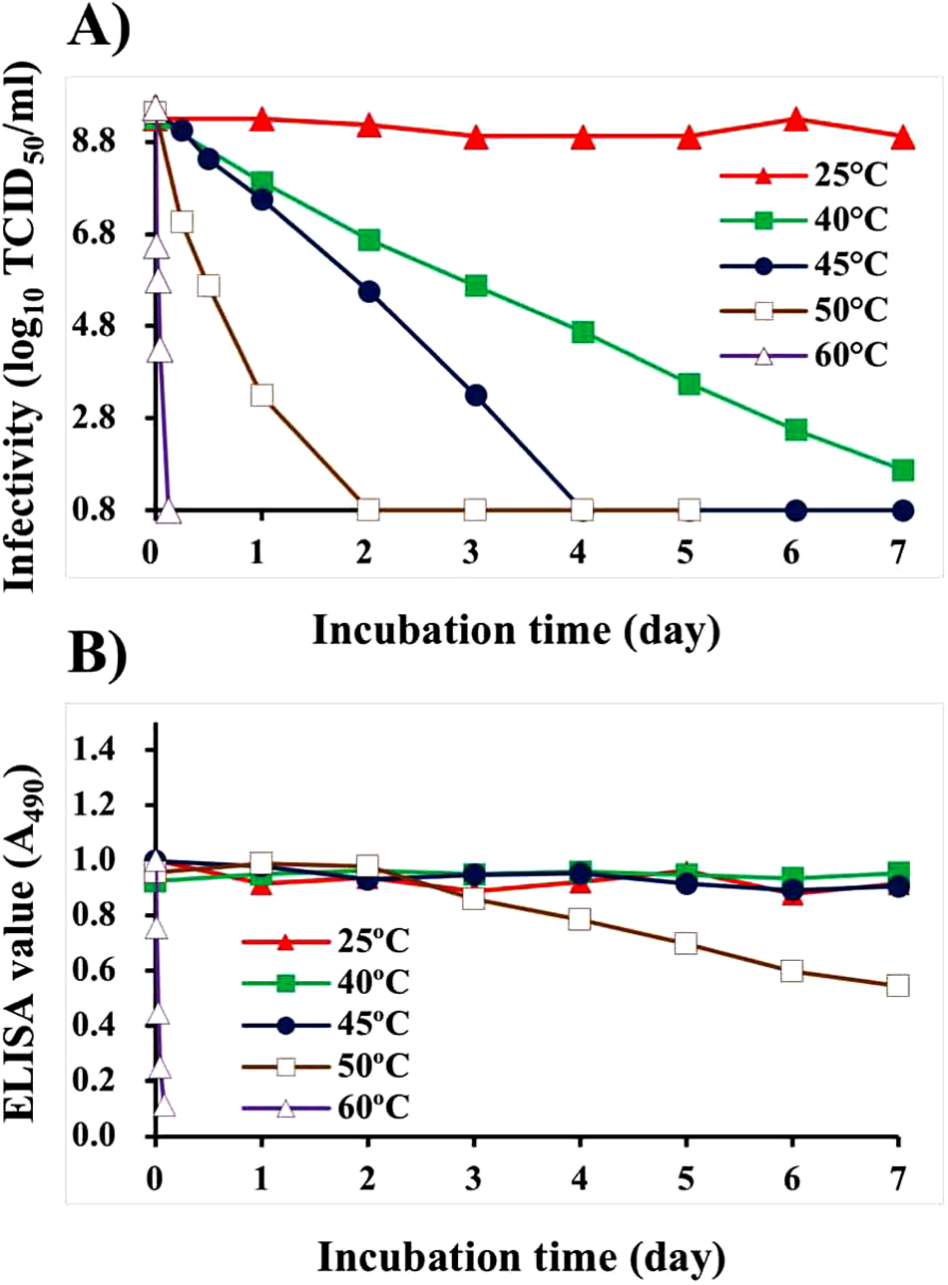
Effect of incubation at different temperatures on infectivity and antigenicity of cultured NNV suspensions. Cultured NNV suspensions were incubated at 25 °C, 40 °C, 45 °C, 50 °C, or 60 °C for up to 7 days. After incubation, each sample was subjected to titration of NNV infectivity and ELISA for detecting NNV antigens. (**A**) NNV infectivity, (**B**) NNV antigenicity.

The infectivity titer of cultured NNV suspension (10^9.3^ TCID_50_/ml) was stable at 25 °C for 7 days. However, the infectivity of an NNV suspension incubated at 40 °C gradually declined to 10^1.2^ TCID_50_/ml on day 7 while infectivities of suspensions incubated at 45 °C, 50 °C, and 60 °C declined to below the detection limit (≤10^0.8^ TCID_50_/ml) within 4, 2, and 0.1 days, respectively (Fig. 1A). Enzyme-linked immunosorbent assay (ELISA) of NNV antigens revealed stable values at temperatures ≤45 °C (0.95 ± 0.03) while those incubated at 50 °C began to decline gradually on day 3, reaching 0.54 on day 7. ELISA values of those incubated at 60 °C were drastically reduced to 0.08 within 0.1 days (Fig. 1A).

### Infectivity and antigenicity of cultured NNV suspensions diluted 320-fold after incubation at 25 °C and 45 °C

**Figure 2:**
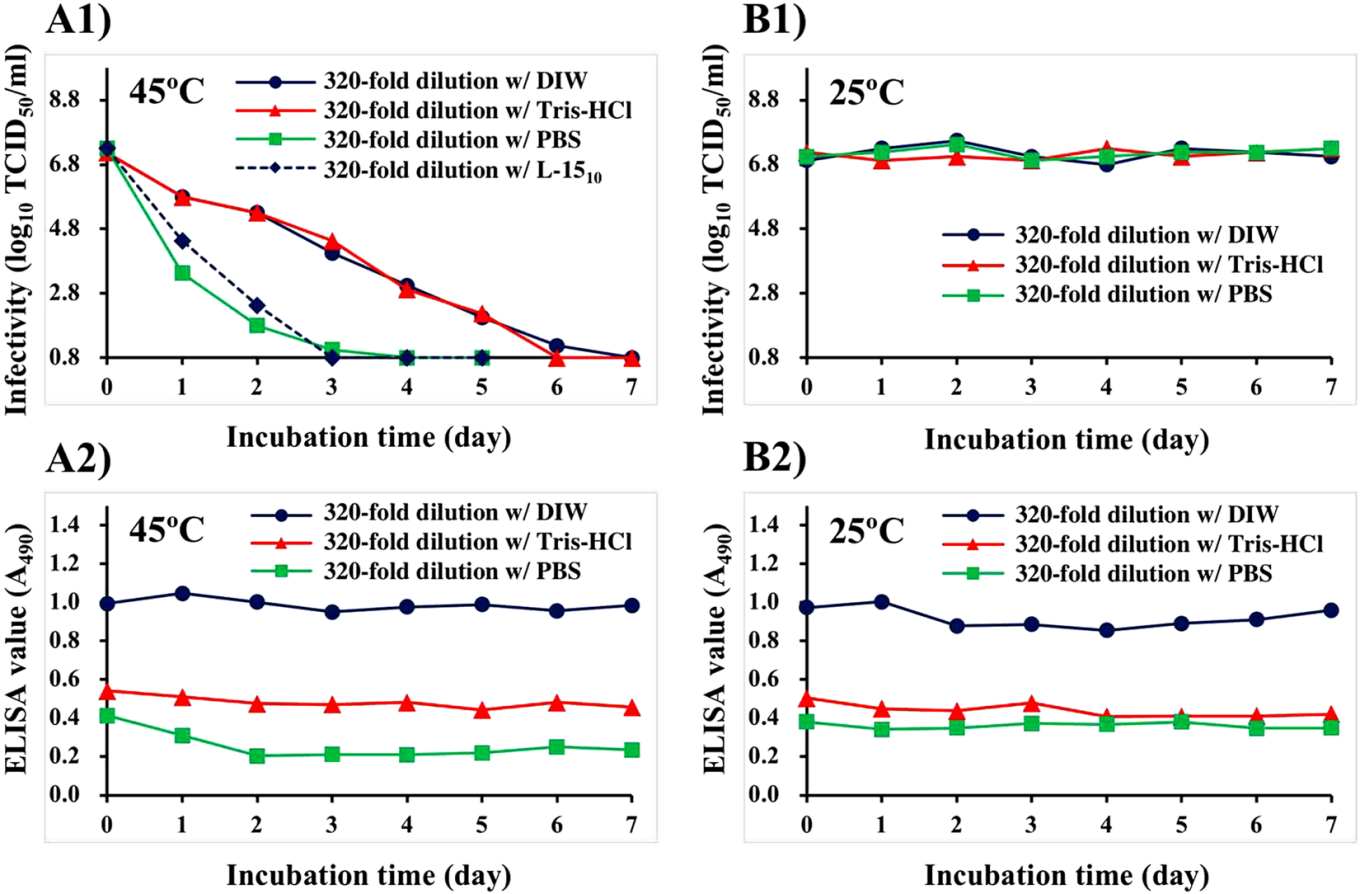
Effect of dilutions with deionized water (DIW), Tris-HCl (15 mM, pH 8.0), or PBS on infectivity and antigenicity of cultured NNV suspensions. Cultured NNV suspension was diluted 320-fold with DIW, 15 mM Tris-HCl (pH 8.0), or PBS, followed by incubation at 25 °C or 45 °C for up to 7 days. (**A**) Incubation at 45 °C, (**B**) Incubation at 25 °C (control). (1) NNV infectivity, (2) NNV antigenicity.

In previous experiments (Fig. 1), cultured NNV suspensions were heat-treated and subsequently diluted 320-fold with deionized water (DIW) followed by immobilization of NNV antigens. In further experiments, cultured NNV suspensions were pre-diluted 320-fold with DIW, Tris-HCl (15 mM, pH 8.0), or Dulbecco’s phosphate buffered saline (PBS) and incubated at 25 °C or 45 °C for 7 days (Fig. 2).

After incubation at 45 °C, infectivity of cultured NNV suspensions diluted with DIW, Tris-HCl (15 mM, pH 8.0), or PBS declined gradually to levels below the detection limit (10^0.8^ TCID_50_/ml) within 4, 6, or 7 days, respectively (Fig. 2A1). The infectivity of cultured suspension diluted with L-15_10_ medium (a control) also declined to levels below the detection limit within 3 days. Following incubation at 45 °C, ELISA values of cultured suspension diluted with DIW (0.99 ± 0.03) were almost unchanged during experiments (Fig. 2A2). However, ELISA values of NNV suspension diluted with Tris-HCl (0.48 ± 0.03) were almost half of those diluted with DIW. NNV suspension diluted with PBS declined from 0.41 to 0.20 after 2 days of incubation at 45 °C. Thereafter, no significant alteration in ELISA value was observed (0.22 ± 0.02) (Fig. 2A2).

Following incubation at 25 °C, the infectivity of cultured suspensions was stable regardless of dilution buffer differences (Fig. 2B1). ELISA values of cultured suspensions diluted with DIW were stable (0.92 ± 0.05) throughout experiments. ELISA values of those diluted with Tris-HCl and PBS (0.44 ± 0.03 and 0.36 ± 0.02, respectively) were almost half of those of NNV suspensions diluted with DIW. These were stable for the duration of experiments (Fig. 2B2).

### Infectivity and antigenicity of purified NNV particles in different buffers

**Figure 3:**
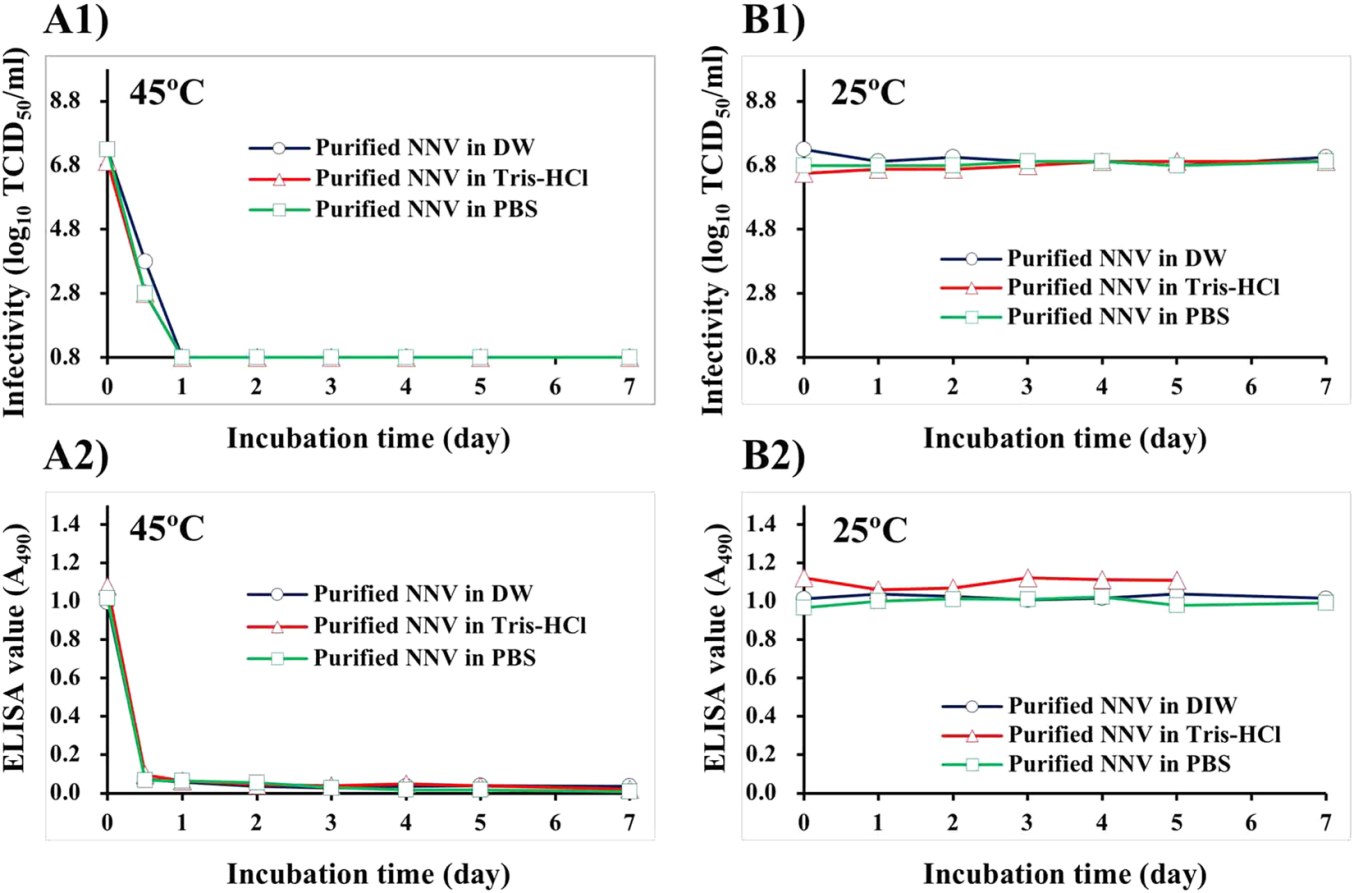
Effect of purified NNV particles suspended in DIW, Tris-HCl (15 mM, pH 8.0), or PBS on infectivity and antigenicity. Purified NNV particles suspended in each buffer were incubated at 45 °C or 25 °C for 7 days. (**A**) Incubation at 45 °C, (**B**) Incubation at 25 °C (control). (1) Alteration in infectivity of purified NNV particles suspended in DIW, Tris-HCl, or PBS, (2) Alteration in antigenicity of purified NNV particles suspended in DIW, Tris-HCl, or PBS.

Next, we were interested in studying the influence of culture medium on NNV behavior. Therefore, we purified NNV particles to see how temperature could affect NNV infectivity and antigenicity in the absence of culture medium components. Following incubation at 45 °C, the infectivity of purified NNV particles decreased to levels below the detectable limit within one day regardless of suspension buffer used (Fig. 3A1). ELISA values of purified NNV particles also decreased to ≤0.07 within half a day (Fig. 3A2).

After incubation at 25 °C, infectivity and ELISA values of purified NNV particles were stable regardless of suspension buffer used (Fig. 3B). Previously, we have shown that the antigenicity of purified NNV particles diminishes after dialysis in PBS at 4 °C due to progressive aggregation of NNV particles^39^. The present study confirmed that antigenicity of NNV particles in PBS decreased at 4 °C, but not at 25 °C (data not shown).

### Effect of 320-fold dilution with DIW, Tris-HCl, or PBS on purified NNV

**Figure 4:**
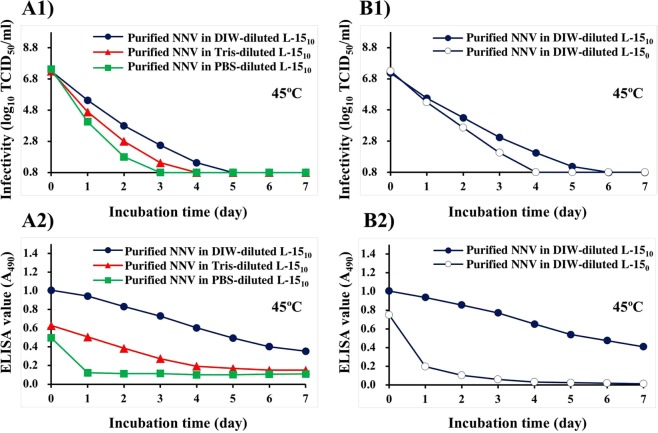
Effect of purified NNV particles suspended in L-15_10_ or L-15_0_ media diluted 320-fold with DIW, Tris-HCl (15 mM, pH 8.0), or PBS. Purified NNV particles suspended in each buffer were incubated at 45 °C for 7 days. (**A**) Purified NNV particles suspended in L-15_10_ media diluted 320-fold with DIW, Tris-HCl, or PBS, (**B**) Purified NNV particles suspended in L-15_10_ or L-15_0_ diluted 320-fold with DIW. (1) Infectivity changes of NNV particles, (2) Antigenicity changes of NNV particles.

The infectivity of purified NNV particles in L-15_10_ media diluted 320-fold with DIW, Tris-HCl (15 mM, pH 8.0), or PBS gradually declined after incubation at 45 °C, reaching levels below the detection limit (≤10^0.8^ TCID_50_/ml) within 5, 4, or 3 days, respectively (Fig. 4A1). ELISA values of purified NNV particles suspended in DIW- or Tris-diluted L-15_10_ media declined gradually from 1.0 to 0.35 or from 0.6 to 0.15 (Fig. 4A2), respectively, whereas those in PBS-diluted L-15_10_ medium declined within 1 day from 0.4 to 0.12 (Fig. 4A2).

### Effect of removing FBS from the culture media

The infectivity of purified NNV particles in DIW-diluted L-15_0_ medium (FBS free) declined to levels below the detection limit within 4 days after incubation at 45 °C whereas that of purified NNV particles in DIW-diluted L-15_10_ medium took two more days to reach levels below the detection limit (Fig. 4B1). ELISA values of purified NNV particles in DIW-diluted L-15_10_ medium declined gradually, reaching 0.41 after 7 days of incubation at 45 °C (Fig. 4B2). Those of purified NNV particles in DIW-diluted FBS-free medium declined from 0.75 to 0.20 within 1 day of incubation at 45 °C. They then gradually declined to 0.01 after 6 more days of incubation (Fig. 4B2).

### Influence of dry-immobilization on NNV antigenicity

**Figure 5:**
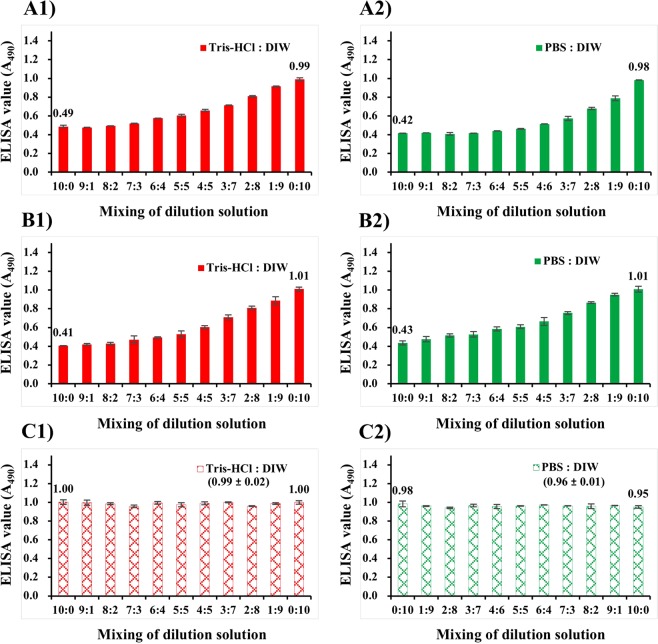
Influence of dry-immobilization on NNV antigenicity. NNV antigens diluted with Tris-HCl or PBS were mixed with those diluted with DIW at different ratios ranging 10:0 to 0:10. These mixed NNV antigens were subsequently immobilized to ELISA plate wells by drying at 37 °C overnight. (**A**) Cultured NNV suspensions diluted with DIW, Tris-HCl, or PBS were mixed at different ratios. (**B**) Purified NNV particles in Tris- or PBS-diluted L-15_10_ media were respectively mixed with those suspended in DIW-diluted L-15_10_ medium at different mixing ratios. (**C**) NNV particles suspended in Tris-HCl or PBS were respectively mixed with those suspended in DIW at different mixing ratios. Error bars indicate standard deviation (SD).

Antigenicity of cultured NNV suspension was almost halved by dilution with Tris-HCl or PBS regardless of incubation temperature (Fig. 2A2,B2). We were interested in studying the influence of salt concentration on NNV antigenicity. Therefore, NNV antigens suspended in different ratio mixtures of Tris-HCl and DIW or PBS and DIW were immobilized. ELISA values of cultured NNV suspension diluted with Tris-HCl (Tris-HCl: DIW at 10:0) or PBS (PBS:DIW at 10:0) were 0.49 or 0.42, respectively (Fig. 5A). These ELISA values increased to 1.0 when mixing ratios of Tris-HCl or PBS were decreased (decreasing in salt concentration). The same results were observed for purified NNV particles suspended in a mixture of Tris-, PBS-, and DIW-diluted L-15_10_ media (Fig. 5B). ELISA values of NNV particles in Tris- or PBS-diluted L-15_10_ (Tris-HCl:DIW or PBS:DIW at 10:0) were 0.41 or 0.43, respectively. These values increased to 1.0 when mixing ratios of Tris-HCl or PBS were decreased (decreasing in salt concentration).

In contrast, for purified NNV particles suspended in Tris-HCl, PBS, or DIW, there was no significant change in ELISA value when mixing ratios of suspension buffers were changed (Fig. 5C). These results demonstrated that salt concentration did not influence antigenicity of NNV particles. However, the antigenicity of NNV particles and/or free-CPs in diluted L-15 medium containing FBS declined with increasing salt concentration regardless of heat-treatment at 45 °C. Such a decline in NNV antigenicity could be due to altered aggregation state of NNV antigens during dry-immobilization, not due to heat-denaturation. FBS and other medium components might play a role in changing aggregation propensity of NNV antigens.

### Focused ion beam scanning electron microscopic (FIB-SEM) visualization of surface protrusions on NNV particles

**Figure 6:**
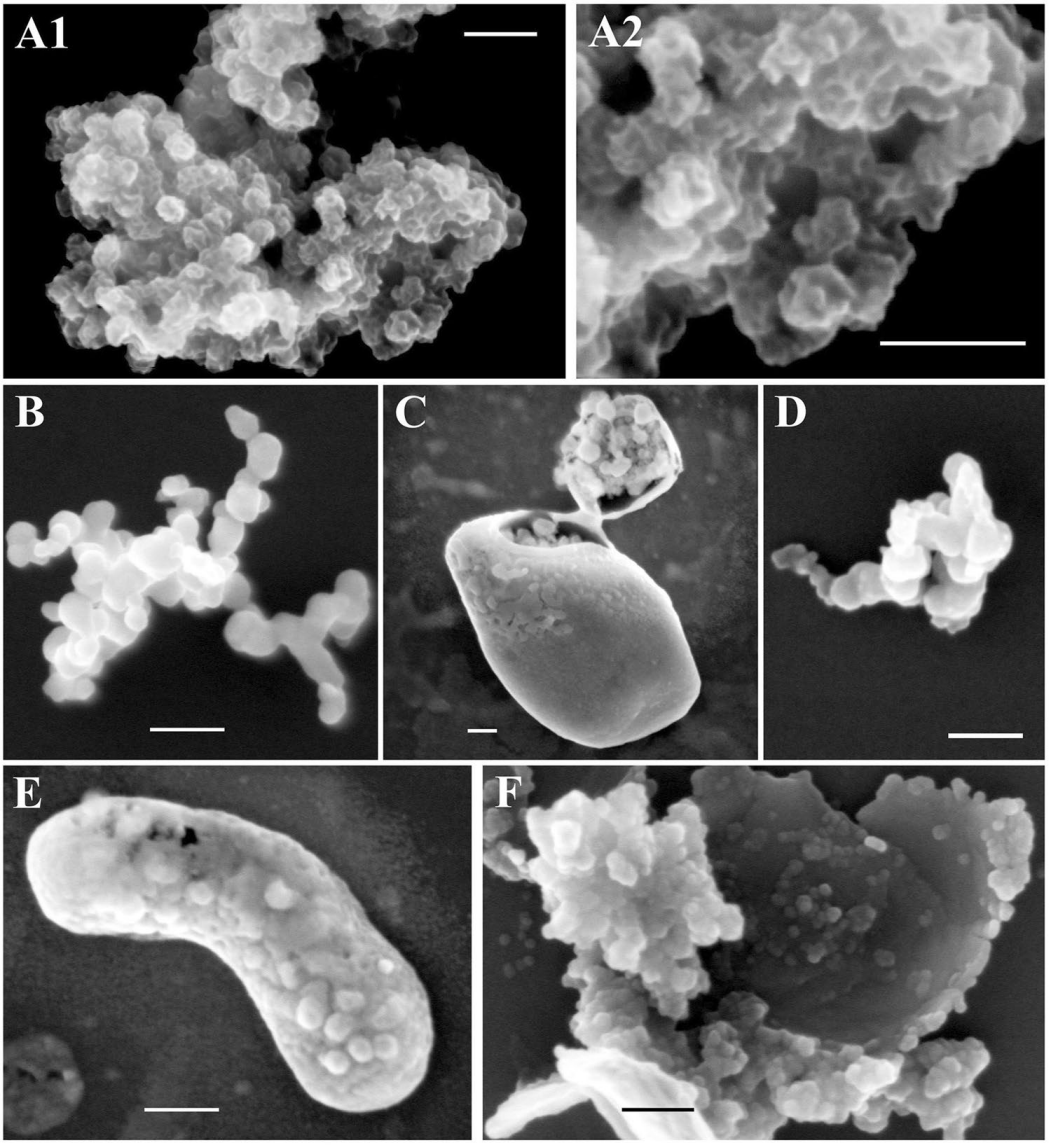
Focused ion beam scanning electron microscope (FIB-SEM) observation of NNV particle’s surface structures. Purified NNV particles were treated at 45 °C for 24 h or at 100 °C for 5 min and subjected to FIB-SEM. (**A1**) and (**A2**) Purified NNV particles without heat-treatment, (**B–C**) Purified NNV particles with heat-treatment at 45 °C for 24 h, (**D–F**) Those treated at 100 °C for 5 min. Scale bars = 100 nm.

Surface structures of purified NNV particles treated at 45 °C for 24 h or at 100 °C for 5 min were imaged using FIB-SEM (Fig. 6). Control samples that were not heat-treated showed NNV particles of approximately 30 nm in diameter. In addition, complex three-dimensional structures on these surfaces were visible (Fig. 6A1–2). However, surface topography completely disappeared after incubation at 45 °C for 24 h (Fig. 6B,C) or at 100 °C for 5 min (Fig. 6D–F) whilst viral particle-like structures with smooth surfaces (32–35 nm in diameter) were reliably present regardless of treatment temperature. It has previously been reported that surface protrusions on NNV particles play a crucial role in viral antigenicity and infectivity^22^ and that these surface protrusions are sensitive to heat-denaturation^37^. Thus, we conclude that complex three-dimensional structures on NNV particles that we observed were surface protrusions (Fig. 6A1,A2). These protrusions were clearly highly susceptible to heat-denaturation while the remaining particle structures were particularly stable.

Interestingly, large aggregates of heat-denatured NNV particles were covered with a thin film-like structures (Fig. 6C,E,F) that could be easily broken by irradiating with a slightly stronger ion beam. There was no contamination of any kind to form thin film-like structures because NNV particles were highly purified^37,39^. Thus, we speculate that these thin structures might form by random re-folding of heat-denatured protrusions together with those of neighboring particles after samples cool down.

## Discussion

The infectivity of cultured NNV suspensions gradually declined following incubation at 45 °C, although its antigenicity was maintained (Fig. 1). In contrast, heating purified NNV at the same temperature caused a loss in both infectivity and antigenicity (Fig. 3A). It has been reported that surface protrusions play a crucial role in viral receptor interactions during NNV infection^22^. By using FIB-SEM, we were able to show that NNV surface protrusions were completely denatured after incubation at 45 °C, although NNV particle structures were reliably preserved (Fig. 6). The NNV antiserum used in this study can recognize heat-sensitive conformational structures of NNV surface protrusions^37^. Therefore, both infectivity and antigenicity of NNV particles should disappear due to heat-denaturation of surface protrusions as shown in Fig. 3A. However, infectivity and antigenicity of cultured NNV behaved differently in response to heating at 45 °C (Fig. 1). A possible explanation lies in the fact that NNV antigens in cultured NNV suspension contain both viral particles and coat proteins not associated with particles (free-CPs). More than 90% of NNV antigens are derived from free-CPs^39^. Furthermore, NNV surface protrusions and free-CPs share common antigens^39^. Therefore, ELISA values with anti-NNV serum in cultured NNV suspension could mainly reflect the antigenicity of NNV free-CPs. In contrast, changes in infectivity directly reflects the denaturation state of surface protrusions. This suggests that conformational structures corresponding to P-domain of NNV free-CPs may be more stable than those of NNV surface protrusions, although they shared some common structural features.

Cultured NNV suspensions diluted with different buffers also showed differences in infectivity and antigenicity following incubation at 45 °C. NNV infectivity declined gradually at 45 °C whereas antigenicity of cultured NNV suspension was relatively stable, especially for DIW-dilution of cultured NNV suspension (Fig. 2A). Interestingly, antigenicities of Tris- or PBS-diluted NNV suspensions were almost half of the antigenicity of DIW-diluted NNV suspension (Fig. 2A2). Such reduction in half for antigenicity was not due to heat-denaturation of NNV free-CPs because the same loss of antigenicity was also observed after incubation at 25 °C (Fig. 2A2). It has been reported that NNV particles and free-CPs in cultured suspension can aggregate together under certain conditions^39^. Furthermore, the antigenicity of these aggregates can change in response to changing salt concentrations as a direct result of differential aggregation properties^40^. The present study provides confirmation that, when cultured NNV suspension is dry-immobilized onto ELISA plate wells after dilution with different mixing ratios of Tris-HCl:DIW or PBS:DIW, NNV antigenicity would decrease with increasing salt concentrations even without heating (Fig. 5A). This is likely due to aggregation-related phenomena.

Infectivity of cultured NNV suspensions diluted with PBS or L-15_10_ medium disappeared within 4 days of incubation at 45 °C whereas virus infectivity with DIW or Tris-HCl disappeared within 7 days (2 to 3 days of delay) (Fig. 2A1). Cultured virus diluted with PBS and L-15_10_ medium were at physiological osmolality (154 mM salts) while those diluted with DIW or Tris-HCl contained approximately 15 mM salts. There was no significant difference in the concentrations of other components. Therefore, salt concentration appears to be important for the stability of NNV surface protrusions.

In contrast, the infectivity of purified NNV particles disappeared within one day following incubation at 45 °C (Fig. 3A1). Interestingly, loss of infectivity of purified NNV particles was delayed 2–4 days after heating in DIW-, Tris-, or PBS-diluted L-15_10_ media (Fig. 4A1). Such delays were more pronounced at lower salt concentration. Furthermore, disappearance of NNV infectivity in DIW-diluted L-15_10_ medium containing FBS was delayed 2 days in comparison with that in DIW-diluted L-15_0_ medium that was FBS free (Fig. 4B1). These results suggest that FBS could function as a conformational stabilizer of NNV surface protrusions (=infectivity). The influence of FBS and/or salt concentration on NNV protrusions was also reproduced by the results of alteration in antigenicity of NNV particles (Fig. 4A2,B2).

As described above, the stability of NNV surface protrusions was apparently related to salt concentration (Figs 2, 4). However, such a relationship was not observed for NNV particles suspended in DIW, Tris-HCl, or PBS (Fig. 3A). To account for the apparent contradiction, we have the following explanations. Fir, the former NNV particles (Figs 2, 4) were suspended in L-15 medium containing FBS whereas the latter NNV particles (Fig. 3A) were suspended in buffer containing no FBS. FBS can function as stabilizer for NNV surface protrusions as described above. Thus, the stabilizing function of FBS could be influenced by salt concentration (i.e., stability of NNV surface protrusions in L-15 medium containing FBS was indirectly influenced by salt concentration through the stabilizing function of FBS). Therefore, no influence of salt concentration on stability of NNV surface protrusions in buffers without FBS was observed (Fig. 3). We speculate that the stabilizing function of FBS might be due to serum albumin, the predominant protein component of FBS. It can serve as a carrier for foreign substances through binding interactions.

Incidentally, as shown in Fig. 4A2, antigenicity of NNV particles suspended in Tris- or PBS-diluted L-15_10_ medium was half of that suspended in DIW-diluted L-15_10_ medium before incubation at 45 °C (on day 0). This reduction in antigenicity did not have any significant influence on infectivity (Fig. 4A1). Furthermore, during dry-immobilization, antigenicity of NNV particles in Tris- or PBS-diluted L-15_10_ medium was halved without heat-treatment (Fig. 5C). Therefore, drops in antigenicity might be due to changes of aggregation states of NNV particles during dry-immobilization, but not due to denaturation of NNV surface protrusions. To minimize these alterations, NNV suspension should be diluted with DIW for ELISA with dry-immobilization of NNV antigens.

In this study, cultured and purified NNV suspensions were treated at moderate-low temperatures to studying stabilities of surface protrusions and free-CPs. These moderate-low temperatures are unrealistic for NNV infection. However, there might be difference in stabilities of conformational structures between P-domain of free-CPs and NNV surface protrusions. These stabilities can change before and after construction of viral particles. We believe that this can be an interesting character for studying assembly and/or uncoating of NNV particles.

## Methods

### Virus and antisera

NNV SgNag05 belonging to RG genotype^28,29^ was cultured with SSN-1 cells at 25 °C. SSN-1 cells were maintained in Leibovitz’s L-15 medium (Gibco) containing 10% (v/v) FBS (Hyclone), 150 IU/ml of penicillin G, and 100 μg/ml of streptomycin. Cultured NNV was centrifuged at 12,000 × g for 20 min at 4 °C. The resulting supernatant was harvested and stored as standardized cultured NNV suspension.

Purification of NNV particles was performed as described previously by Gye and Nishizawa^39,40^. Briefly, using a tube made of Biotech cellulose ester (CE) membrane with a molecular weight cut off (MWCO) of 10^6^ (Spectrum Laboratories), standardized cultured NNV suspension was dialyzed in PBS for 1 day, 15 mM Tris-HCl (pH 8.0) for 3 days, and DIW for 1 day. The dialyzed NNV suspension was then subjected to anion-exchange chromatography using a Hi-trap Q column (GE Healthcare). Fractions of chromatogram peaks eluted with 700 mM NaCl were collected. These eluted NNV particles were then washed with DIW using centrifugal ultrafiltration (10^4^ MWCO, Vivaspin, Sartorius) according to the manufacturer’s instructions.

NNV infectivity was titrated using 96-well microplates seeded with SSN-1 cells. Appearance of cytopathic effect (CPE) was evaluated to determine 50% tissue culture infectious dose (TCID_50_) after 10 days of culture at 25 °C. Infectivity titers for cultured and purified NNV suspensions were approximately 10^9.3^ and 10^8.3^ TCID_50_/ml, respectively.

Anti-NNV serum was previously prepared in our laboratory^41^. In brief, FPLC purified NNV particles emulsified with Freund’s incomplete adjuvant (Sigma-Aldrich) were injected into a New Zealand white rabbit. The rabbit was reinjected intravenously with purified NNV particle suspension three times with 10-day intervals as boosters. Final bleeding was performed at 3 days after the 4th injection. This experimental protocol was approved by the Institutional Animal Care and Use Committee of Chonnam National University (Approval No: CNU IACUC-YS-2015–3), and all experiments were performed in accordance with relevant guidelines and regulations. It has been reported that this anti-NNV serum can recognize heat-sensitive conformational structures of protrusions on NNV particle surface^37^.

### Heat-treatment of cultured NNV suspension without dilution

Cultured NNV suspension was incubated at 25 °C, 40 °C, 45 °C, 50 °C, or 60 °C for up to 7 days. Each heat-treated NNV suspension was subjected to titration of NNV infectivity and ELISA to detect NNV antigens. Prior to be their use as ELISA antigens, cultured NNV suspensions after heat-treatments were diluted 320-fold with DIW because adsorption of NNV antigens to ELISA plates could be inhibited by FBS and other components present in the culture medium^42^.

### Heat-treatment of cultured NNV suspension diluted with different buffers

Cultured NNV suspension was diluted 320-fold with DIW, 15 mM Tris-HCl (pH 8.0), or PBS and incubated at 25 °C or 45 °C for up to 7 days. These heat-treated samples were subjected to titration of NNV infectivity and ELISA to detect NNV antigens. As a control, cultured NNV suspension was diluted 320-fold with L-15 medium containing 10% FBS (L-15_10_) for titration of NNV infectivity. However, this sample was not used as an ELISA antigen due to inhibition problem caused by culture medium components as described above.

### Heat-treatment of purified NNV particles in different buffers

Purified NNV particles were suspended in DIW, Tris-HCl (15 mM, pH 8.0), or PBS and incubated at 25 °C or 45 °C for 7 days. These heat-treated samples were subjected to titration of NNV infectivity and ELISA to detect NNV antigens.

### Influence of heat-treatment on infectivity and antigenicity of purified NNV particles in L-15_10_ media diluted with different buffers

Assuming the same experimental conditions as shown in Fig. 2, L-15_10_ medium was diluted 320-fold with DIW, Tris-HCl (15 mM, pH 8.0), or PBS to suspend purified NNV particles. In addition, L-15 medium without FBS (L-15_0_) was diluted 320-fold with DIW to suspend purified NNV particles. These purified NNV particles in each solution were incubated at 45 °C for 7 days and subsequently subjected to titration of NNV infectivity and ELISA to detect NNV antigens.

### Influence of dry-immobilization on NNV antigenicity

Cultured NNV suspension was diluted 320-fold with DIW, Tris-HCl (15 mM, pH 8.0), or PBS (under the same conditions as shown in Fig. 2). These NNV suspensions diluted with Tris-HCl and PBS were then respectively mixed with those diluted with DIW at different mixing ratios from 10:0 to 0:10. Without heat-treatment at 45 °C, these mixed NNV suspensions were subsequently immobilized to ELISA plates by drying at 37 °C overnight to detect NNV antigens.

L-15_10_ medium was diluted 320-fold with DIW, Tris-HCl (15 mM, pH 8.0), or PBS. Purified NNV particles were then suspended in diluted L-15_10_ media as described above. NNV particles suspended in Tris- or PBS-diluted L-15_10_ media were respectively mixed with DIW-diluted L-15_10_ medium at different mixing ratios and subsequently immobilized to ELISA plates by drying at 37 °C.

Purified NNV particles were suspended in Tris-HCl (15 mM, pH 8.0), PBS or DIW. These NNV particles in Tris-HCl or PBS were respectively mixed with those suspended in DIW at different mixing ratios and subsequently dry-immobilized to ELISA plates.

### Enzyme-linked immunosorbent assay (ELISA) for detecting NNV antigens

ELISA for detecting NNV antigens was performed following published methods^37,41^. In brief, NNV suspension was placed into wells of ELISA plates (Greiner bio-one) at 100 μl/well and fixed by drying at 37 °C overnight. Wells of ELISA plates were blocked with 5% skim milk in PBS (SM-PBS) at 25 °C for 30 min. After washing three times with PBS, anti-NNV rabbit serum diluted with SM-PBS was added into each well and incubated at 25 °C for 30 min. Antibodies bound to NNV antigens fixed on ELISA plates were detected using horseradish peroxidase (HRP) conjugated antiserum against rabbit Ig (Dako, diluted with SM-PBS) and OPD substrate solution (1 mg/ml *ο*-phenylenediamine, 0.03% H_2_O_2_, 100 mM Na_2_HPO_4_, 50 mM citric acid). The reaction was halted by adding 100 μl of 2N H_2_SO_4_. Absorbance value at wavelength 490 nm (A_490_) was obtained using a microplate reader (SpectraMax™ 340PC^384^, Molecular Devices). Data are presented as averages of duplicate wells.

### Focused ion beam scanning electron microscope (FIB-SEM)

Purified NNV particles with or without heat-treatments were placed onto the sample stage of FIB-SEM and immobilized by drying at room temperature. These NNV particles on the sample stage were observed with an FIB-SEM (Helios NanoLab G3, FEI^TM^) according to the manufacturer’s instructions.
